# Significant neutralizing activity of human immunoglobulin preparations against pandemic 2009 H1N1

**DOI:** 10.1111/j.1365-2141.2009.08016.x

**Published:** 2009-11-24

**Authors:** Mikihiro Yunoki, Ritsuko Kubota-Koketsu, Takeru Urayama, Tadahiro Sasaki, Du Analiwa, Yuko Konoshima, Shoji Ideno, Yuki Fukunaga, Saeko Morikawa, Satoshi Hiroi, Kazuo Takahashi, Yoshinobu Okuno, Katsuro Hagiwara, Kazuyoshi Ikuta

**Affiliations:** 1 Osaka Research Laboratory, Benesis CorporationOsaka; 2 Department of Virology, Research Institute for Microbial Diseases, Osaka UniversityOsaka; 3 Department of Infectious Diseases, Osaka Prefectural Institute of Public HealthOsaka; 4 Kanonji Institute, The Research Foundation for Microbial Diseases of Osaka UniversityKagawa; 5 School of Veterinary Medicine, Rakuno Gakuen UniversityHokkaido, Japan

The influenza-like illness that began in the Unites States and Mexico was first reported by the World Health Organization (WHO) on 24 April, 2009, and declared a phase 6 pandemic on 11 June. As of 6 July 2009, over 90 000 cases and more than 400 deaths in some 120 countries had been confirmed (WHO, 2009). Importantly, on July 8th, the WHO announced that oseltamivir (Tamiflu)-resistant viruses had been identified in Denmark, Japan and Hong Kong (WHO, 2009).

The pandemic virus 2009 H1N1 was a triple reassortant of human-, swine- and avian-derived influenza A virus segments and the HA gene was classified as being of swine-origin (Novel Swine-Origin Influenza A (H1N1) Virus Investigation Team, (2009). Evidence is accumulating that specific IgG antibodies against this virus are present in certain populations, especially the elderly (Itoh *et al*, 2009). However, Katz *et al* (2009) reported that cross reactive IgG against a pandemic influenza virus (A/California/04/2009) was found in no serum specimens of children aged 6 months–9 years old, 8% of samples from 5- to 9-year olds, 9% of samples from 18- to 64-year olds, 6% of samples of 18- to 40-year olds and 33% of samples of those over 60 years old, suggesting that immunoglobulin preparations derived from pooled plasma from over 10 000 healthy donors could contain such cross reactive IgG. The present study evaluated haemagglutinin-inhibition (HI) and virus neutralization (VN) activities against 2009 H1N1 and seasonal H1N1 as a positive control in intravenous human immunoglobulin (IVIG) preparations manufactured in 1999 and 2008.

An influenza A/H1N1 vaccine strain (A/New Caledonia/20/99), a clinical isolate of A/H1N1 (A/Osaka/16/2008), a classical swine isolate of A/H1N1 (A/Swine/Hokkaido/2/1981) and a pandemic influenza isolate of A/H1N1 (A/Osaka/168/2009 H1N1 pdm) were used in this study. Three lots (Lot. A, B and C) of IVIG derived from pooled plasma collected in Japan and manufactured in 2008 (IVIG2008JP, ‘Kenketsu Venoglobulin®-IH Yoshitomi; Benesis Corp., Osaka, Japan’) were also used. In addition, two lots of IVIG that were manufactured in 1999, derived from plasma pooled collected in Japan and the USA (IVIG1999JP ‘Kenketsu Venoglobulin®-IH’, IVIG1999US ‘Venoglobulin®-IH; Yoshitomi Pharmaceutical Industries, Ltd. at the time, currently Benesis Corp.’), were used.

The viruses were propagated in Madin-Darby canine kidney (MDCK) cells or in the allantoic cavity of chicken embryonated eggs. The culture media and the allantoic fluids were stored at −80°C prior to use. Infectivity, as infectious focus-forming units (FFU) per ml, was titrated in MDCK cells using peroxidase and an anti-peroxidase (PAP) staining technique (Okuno *et al*, 1990). The haemagglutinin-inhibition (HI) test using 0·75% guinea pig red blood cells was carried out as described previously (Okuno *et al*, 1993). The results were expressed as the reciprocal of the highest dilution of the culture medium to show inhibition. The virus neutralization (VN) test was carried out as described (Okuno *et al*, 1990). Briefly, IVIG was diluted twofold with serum-free medium. The diluted IVIG (50 μl) was mixed with 100 FFU (50 μl) of virus, then applied to MDCK cells in a 96-well microplate. After culturing, the cells were fixed with ethanol and stained by PAP as above. The results were expressed as the reciprocal of the dilution giving 50% neutralization.

Intravenous human immunoglobulins were manufactured using plasma pooled from over 10 000 healthy donors. The HI and VN activities of IVIGs were titrated against pandemic, seasonal human and swine influenza A viruses (Table I). Of note, both the 1999 and 2008 IVIGs were shown to have anti pandemic and classical swine influenza A/H1N1 virus titres with HI (×4–×8) and VN (×32–×64). The 2008 IVIGs showed titres against the vaccine strain A/New Caledonia/20/99, which was isolated in 1999, with HI (×160–×320) and VN (×640–×1280), while the 1999 IVIGs showed titres with HI (×10–×40) and VN (×32–×128). These results suggested that the IVIG derived from the pooled plasma contained a certain amount of functional IgG, including IgG against pandemic or classical swine influenza A/H1N1. Of note, such IgG titres were slightly higher in the IVIG2008JP products compared with IVIG1999JP. However, the titres were slightly higher in IVIG1999US than in IVIG1999JP. Higher titres against the vaccine and clinical strains were observed in IVIG1999US than IVIG1999JP. Interestingly, the difference in the increase in titres against the vaccine strain was much greater between the products manufactured in 2008 and 1999 than between the others. This difference seems to be an outcome of vaccination. Our preliminary results showed a HI titre >×40 in 1·2% (7/580), ×20 in 3·1% (18/580) and ×10 in 4·3% (25/580), indicating the possible production of hyperimmune globulin with these sources of plasma collected in 2008, Japan.

**Table I:** Cross reactivity of several lots of IVIG against pandemic 2009, classical swine and seasonal H1N1 viruses.

	Pandemic 2009 H1N1 A/Osaka/168/ 2009(H1N1pdm)		Classical swine H1N1 A/Swine/ Hokkaido/2/1981		Vaccine strain H1N1 A/NC/20/99		Clinical isolate H1N1 A/Osaka/16/2008	
IVIG	HI	VN	HI	VN	HI	VN	HI	VN
2008JP, Lot. A	8	64	8	64	160	640	20	160
2008JP, Lot. B	8	64	8	64	160	640	20	160
2008JP, Lot. C	8	64	8	64	320	1280	40	160
1999US, Lot. D	16	64	16	64	40	128	16	32
1999JP, Lot. E	8	32	4	64	10	32	4	8

JP, Japan; US, United States; HI, haemagglutinin-inhibition; VN, virus neutralization.
